# Antibody-dependent immune responses elicited by blood stage-malaria infection contribute to protective immunity to the pre-erythrocytic stages

**DOI:** 10.1016/j.crimmu.2022.100054

**Published:** 2022-12-23

**Authors:** Irene Tumwine-Downey, Katrien Deroost, Prisca Levy, Sarah McLaughlin, Caroline Hosking, Jean Langhorne

**Affiliations:** Malaria Immunology Laboratory, Francis Crick Institute, London, NW1 1AT, UK

## Abstract

Advances in transcriptomics and proteomics have revealed that different life-cycle stages of the malaria parasite, *Plasmodium,* share antigens, thus allowing for the possibility of eliciting immunity to a parasite life-cycle stage that has not been experienced before. Using the *Plasmodium chabaudi* (AS strain) model of malaria in mice, we investigated how isolated exposure to blood-stage infection, bypassing a liver-stage infection, yields significant protection to sporozoite challenge resulting in lower liver parasite burdens. Antibodies are the main immune driver of this protection. Antibodies induced by blood-stage infection recognise proteins on the surface of sporozoites and can impair sporozoite gliding motility *in vitro,* suggesting a possible function *in vivo.* Furthermore, mice lacking B cells and/or secreted antibodies are not protected against a sporozoite challenge in mice that had a previous blood-stage infection. Conversely, effector CD4^+^ and CD8^+^ T cells do not seem to play a role in protection from sporozoite challenge of mice previously exposed only to the blood stages of *P. chabaudi*. The protective response against pre-erythrocytic stages can be induced by infections initiated by serially passaged blood-stage parasites as well as recently mosquito transmitted parasites and is effective against a different strain of *P. chabaudi* (CB strain), but not against another rodent malaria species, *P. yoelii*. The possibility to induce protective cross-stage antibodies advocates the need to consider both stage-specific and cross-stage immune responses to malaria, as natural infection elicits exposure to all life-cycle stages. Future investigation into these cross-stage antibodies allows the opportunity for candidate antigens to contribute to malaria vaccine development.

## Highlights

• Immunity to pre-erythrocytic parasites induced by malaria blood-stage infection.
• Protective immunity is mediated by B cells and antibodies.
• Immunity is effective against another strain of *Plasmodium chabaudi.*
• Antibodies induced by blood stages recognise antigens on the sporozoites surface.
• Cross-stage reactive antibodies impair sporozoite gliding motility.

## Graphical abstract

**Figure:**
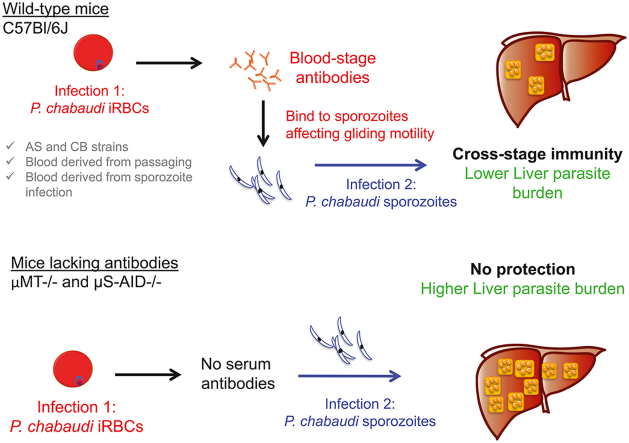

## 1 Introduction

The malaria parasite, *Plasmodium,* has a complex life cycle that involves stages in the mosquito vector and the mammalian host, which can be broadly categorised into three phases: i) the pre-erythrocytic stages, including sporozoites and liver-stage parasites, ii) the erythrocytic stages comprising the asexual and gametocyte stages within red blood cells in the mammalian host, and iii) the sexual stages within the mosquito vector giving rise to infective sporozoites. In 2020, this complex parasitic infection led to 241 million cases of malaria with 627,000 deaths globally (World Health Organization, 2021). Individuals in endemic areas can develop naturally acquired immunity to malaria, however this is slow to develop, requires continued exposure and does not prevent the acquisition and further transmission of the parasite (Diperri et al., 1995; Barry and Hansen, 2016). Thus, understanding the mechanisms underpinning protection to each of the life-cycle stages in the mammalian host is vital for successful vaccine development.

*Plasmodium* life-cycle stages and their corresponding immune responses have mostly been studied in isolation, whereby the host is immunised with antigens from one life-cycle stage, and then challenged with parasites of the same stage, or exposed to the full mammalian infection cycle (Langhorne et al., 2008; Doolan et al., 2009; Renia and Goh, 2016; Long and Zavala, 2017; Kurup et al., 2019; Perez-Mazliah et al., 2020). Thus, it has been assumed that immunity to malaria is mostly stage-specific, and vaccines have been designed accordingly (Duffy and Patrick Gorres, 2020). However, the majority of challenge experiments do not investigate stage-transcending immunity, even though within the large repertoire of antigens expressed by *Plasmodium*, some are shared across life-cycle stages (Hall et al., 2005; Tarun et al., 2008; Howick et al., 2019).

Many experimental vaccination models use whole sporozoites, which have been irradiated (Nussenzweig et al., 1967; Seder et al., 2013), genetically attenuated (Spring et al., 2013; Mueller et al., 2005) or administered under drug cover (Beaudoin et al., 1977; Mordmuller et al., 2017) to generate pre-erythrocytic stage immunity (Bijker et al., 2015; Goh et al., 2019). Infection with live sporozoites under malaria chemoprophylaxis (CPS, chemoprophylaxis and sporozoites) is a very effective means of immunising humans (Roestenberg et al., 2009, 2011; Behet et al., 2014) and mice (Beaudoin et al., 1977; Orjih et al., 1982; Belnoue et al., 2004) against challenge with *Plasmodium*. It is generally believed the protective immunity generated in these models is directed at the pre-erythrocytic stages, as the chemoprophylaxis used during immunisations limits erythrocytic-stage cycles and immunised individuals have been reported not to display immunity to direct blood-stage challenge (Bijker et al., 2013). However, some studies show CPS can induce some protection against direct blood-stage challenge (Inoue et al., 2012; Peng et al., 2014; Doll et al., 2014; Nahrendorf et al., 2015). In those experiments, it was not possible to distinguish between an immune response to antigens shared between pre-erythrocytic and erythrocytic stages, or to antigens expressed on the very limited blood cycle that takes place after chemoprophylaxis. By contrast, we and others have demonstrated cross-stage immunity to pre-erythrocytic stages induced by exposure to blood-stage parasites, bypassing exposure to preceding pre-erythrocytic parasites; (Belnoue et al., 2008; Nahrendorf et al., 2015; Demarta-Gatsi et al., 2016; Lu et al., 2017).

Here we have investigated the nature of protective immunity to pre-erythrocytic stages of *P. chabaudi* that can be induced by isolated exposure to blood-stage parasites. We show that partial immunity is elicited, which is species- but not strain-specific and can be induced even in the presence of an ongoing low-level blood-stage infection. This cross-stage immunity against pre-erythrocytic stages of *P. chabaudi* is mediated by antibodies, which we could demonstrate bind to the surface of sporozoites and inhibit the gliding motility of sporozoites *in vitro*, alluding to a protective mechanism. Utilising these cross-stage antibodies for the identification of *Plasmodium* antigens shared across different life-cycle stages would be a useful addition to the repertoire of potential anti-malaria vaccines.

## 2 Methods

### 2.1 Mice

Wildtype C57Bl/6J, immunoglobulin μ-chain knockout mice, B6.μMT−/− (Kitamura et al., 1991) and secretory -chain (μS)/activation-induced cytidine deaminase (AID) double-knockout mice, μS−/−AID−/− (Kumazaki et al., 2007) mice were bred at the Francis Crick Institute under specific pathogen-free conditions. Mice were housed under reverse light conditions (light 19.00–07.00, dark 07.00–19.00 GMT) at 20–22 °C for a minimum of one week prior to use. Mice were given water and diet *ad libitum*. All procedures were carried out in accordance with the UK Animals (Scientific Procedures) Act 1986, under the Home Office licences 80/2538, 70/8326 and PADD88D48. Female mice aged 6–10 weeks were used for all studies. Age-matched female C57Bl/6/J mice were used as controls in all experiments.

### 2.2 Parasites and blood-stage *Plasmodium* infections

Cryopreserved cloned lines of *Plasmodium chabaudi chabaudi* AS strain and *Plasmodium chabaudi chabaudi* CB strain were originally obtained from Professor David Walliker, University of Edinburgh, and *Plasmodium yoelii* 17XL strain was obtained from Professor Anthony Holder, Francis Crick Institute. Serially blood-passaged (SBP) infections were derived from a cryopreserved stock of infected blood and maintained through passaging in mice, as previously described (Spence et al., 2013; Ogun et al., 2011). Recently mosquito-transmitted (RMT) *P. chabaudi* infected red blood cells (iRBCs) were derived from mice that had been infected by the bites of infected mosquitoes as described (Spence et al., 2012). Blood-stage *Plasmodium* infections were initiated in mice by intraperitoneal (i.p) injection of 10^5^ iRBCs. *Plasmodium chabaudi chabaudi* AS strain parasites (*P. chabaudi* AS, RMT) were used in most experiments unless otherwise stated.

### 2.3 Mosquitoes and pre-erythrocytic stage *Plasmodium chabaudi* infections

*Anopheles stephensi* mosquitoes (SD500 strain) were bred and infected as previously described with some modifications (Spence et al., 2012). In summary, female mosquitoes were fed with defibrinated horse blood (E & O Laboratories Ltd) via a Hemotek® membrane feeding system to produce eggs. Eggs, larvae and pupae were all maintained in 0.3 g/L sea salt in reverse osmosis water. Larvae were fed with Liquifry No. 1 Food for Baby Egg Laying Fish (Interpet) and then Nishikoi® growth food for Koi and Pond fish, medium pellets. From emergence, adults were fed with sterile filtered 8% (w/v) D-Fructose (Sigma) and 0.05% (w/v) para-aminobenzoic acid (Sigma) in deionized water continuously, with feed replaced on alternate days. All stages were maintained at 28 °C and 75% humidity. Mosquitoes were infected with *P. chabaudi* AS via blood feeding with gametocyte donor mice, as described (Spence et al., 2012). Infected mosquitoes were maintained at 26 °C and 75% humidity. Day 6 post blood feed, mosquitoes received a second feed with uninfected mice. From day 15 post gametocyte feed, salivary glands were isolated via microdissection and sporozoites were released and enumerated. Pre-erythrocytic stage infections in mice were initiated with either 20 infected mosquito bites or 100 sporozoites via intravenous (i.v) tail injection as described (Spence et al., 2012). Mosquitoes were also infected with mCherry-expressing *P. chabaudi* AS parasites (Brugat et al., 2017a) to produce mCherry-expressing sporozoites.

### 2.4 Infection with *P. chabaudi* or *P. yoelii* infected red blood cells*,* and challenge with *P. chabaudi* sporozoites

C57BL/6J mice were infected with *P. chabaudi* blood-stage parasites as described above and challenged with sporozoites via infected mosquito bites (Nahrendorf et al., 2015). Control mice only received sporozoites. Alternatively, C57BL/6J mice were infected with *P. chabaudi* AS, CB or *P. yoelii* blood-stage parasites as described above, and then treated with chloroquine (Sigma) 25 mg/kg body weight in a sterile 0.9% sodium chloride solution on days 24–28 (Achtman et al., 2007) to eliminate any residual blood-stage parasites. On day 48 post infection mice were challenged with 100 *P. chabaudi* sporozoites via i.v tail injection as above. In experiments where knock-out mice were used, all mice (including controls) were treated with chloroquine on days 24–28 to eliminate the blood-stage infection as described above (Achtman et al., 2007). Blood-stage parasitaemias were monitored by Giemsa-stained thin blood smears.

### 2.5 *In vivo* cell depletions and flow cytometry

C57BL/6J mice were treated with either 0.35 mg of anti-CD4 monoclonal antibody (clone YTS 191, BioXcell), 0.1 mg anti-mouse CD8β monoclonal antibody (Lyt 3.2, BioXcell) or 0.85 mg of isotype control rat IgG2b (clone TNP6A7, BioXcell). Doses were given on day −3, −1 prior to challenge and day +1 post sporozoite challenge, 100 μL i.p. To assess the efficacy of cell depletion, single-cell suspensions from perfused livers were obtained as previously described (Mastelic et al., 2012; Tupin and Kronenberg, 2006). Cells were stained with the following antibodies: Brilliant Violet 421™ anti-mouse CD3 (1 in 200 dilution, Biolegend®), PE anti-mouse CD8 (1 in 400 dilution, Biolegend®), APC anti-mouse CD4 (1 in 400 dilution, Biolegend®) and the Fixable Blue Dead cell stain kit (1 in 200 dilution, Invitrogen™, L34961). Cells were acquired using a BD LSRFortessa™ flow cytometer and BD FACSDIVA™ software. Data were analysed using FlowJo™ software (Tree Star).

### 2.6 Quantification of *P. chabaudi* parasites in the liver

Mice were given terminal anaesthesia and were perfused intracardially with 5–10 mL 1X phosphate-buffered saline, pH 7.4 (PBS, Gibco™). Livers were homogenised in 4 mL of TRI Reagent® (Ambion™) in gentleMACS™ M Tubes on a gentleMACS™ Octo Dissociator (Miltenyi Biotec) using programme RNA-01. Three 1 mL aliquots per liver were frozen immediately on dry ice and stored at −80 °C. One 1 mL aliquot per sample was used for RNA isolation, using the Ambion™ RiboPure™ RNA Purification Kit (Invitrogen™) according to the manufacturer's instructions. cDNA was synthesised with 500 ng RNA and *P. chabaudi*-specific 18s rRNA primers and standards were used in quantitative real-time PCR assays (Spence et al., 2012; Nahrendorf et al., 2015). Each qPCR reaction consisted of 1X TaqMan™ fast advanced mastermix (Applied Biosystems™), 300 nM forward primer (5′-AAGCATTAAATAAAGCGAATACATCCTTAT-3′), 300 nM reverse primer (5′GGGAGTTTGGTTTTGACGTTTATGCG-3′), 250 nM probe ([6FAM]CAATTGGTTTACCTTTTGCTCTTT[TAM]) and 2 μL of cDNA (cDNA diluted 1 in 20 in Nuclease-free water (Ambion™)). Samples were run on a QuantStudio™ 3 Real-Time PCR machine (Applied Biosystems™) on the following programme: 50 °C for 2 min, 95 °C for 20 s, followed by 40 cycles of 95 °C for 1 s and 60 °C for 20 s. Parasite copy number was calculated using standard curve quantification as previously described (Nahrendorf et al., 2015). Liver parasite burdens were presented as copy number per whole liver, or as a percentage of the challenge infection controls (mice that only received the sporozoites).

### 2.7 Parasite lysates and preparation of sera

*P. chabaudi* SBP blood-stage lysates were generated as previously described (Quin and Langhorne, 2001, Brugat et al., 2017b). Sporozoite-stage lysate was prepared by first gradient purifying day 15 *P. chabaudi* sporozoites using Accudenz (Accurate Chemical) as previously described (Kennedy et al., 2012). Isolated sporozoites were lysed with a lysis buffer containing 0.4% w/v sodium dodecyl sulphate (SDS), 4 M urea, 20 mM Tris-HCl pH 8.0) and 1X protease inhibitors (Swearingen et al., 2016). Protein concentrations were determined using the Pierce™ BCA Protein Assay Kit according to the manufacturer's instructions (Thermo Scientific™). Sera were prepared from blood taken from mice day 50 or day 100 post blood-stage infection, day 50 or day 100 post mosquito bite infection, or from uninfected naïve mice. Blood was allowed to clot at room temperature for 30 min. Samples were centrifuged at 1000×*g* for 10 min at 4 °C and serum (supernatant) was collected.

### 2.8 ELISA assays

ELISAs to measure the IgG antibodies binding to *P. chabaudi* parasite lysates were performed as previously described with minor modifications (Langhorne et al., 1989; Spence et al., 2013). In brief, Nunc PolySorb™ flat-bottom 96-well plates (Thermo Scientific™) were coated with a blood-stage or sporozoite lysate (5 μg/mL in PBS) prepared as described above. To capture *P. chabaudi-*specific IgG a goat anti-mouse IgG (H + L) antibody conjugated to alkaline phosphatase (1 in 100 dilution, Southern Biotech) was used as the detection antibody. Alkaline phosphatase was detected with 1 mg/mL p-nitrophenyl phosphate substrate (Sigma). Optical density (OD) was measured at 405 nm using a Tecan Safire 2™ microplate reader and Magellan™ software. *P. chabaudi* specific IgG was measured relative to a standard hyperimmune blood-stage serum as described (Li and Langhorne, 2000; Spence et al., 2013). Data were presented as OD values or as arbitrary units (AU) calculated from the standard curve.

### 2.9 Immunofluorescence and imaging cytometry

Thin blood smears were prepared from peripheral blood of C57BL/6J mice at day 7 post *P. chabaudi* AS blood-stage infection, and salivary gland sporozoites taken from day 15 of a mosquito infection were fixed onto microscope slides with methanol: acetone (1:1, VWR) and permeabilised with 0.5% Triton X-100 in PBS (Invitrogen™). Blood smears and sporozoites were incubated with either i) serum obtained from mice that were at day 100 post blood-stage infection initiated by iRBCs, ii) serum from naïve mice or iii) no serum (1 in 100 dilution, 10% FCS in PBS) for 1 h at room temperature. Samples were stained with Alexa Fluor™ 594 goat anti-mouse IgG antibodies at room temperature for 1 h (1 in 500, Invitrogen™). Cells were mounted with media containing DAPI to stain parasite nuclei (Vectashield® plus antifade mounting medium with DAPI, VectorLabs). Cells were imaged on a Zeiss Axioimager fluorescence microscope using the 63X oil objective. Images were analysed using the Fiji application (Schindelin et al., 2012). To assess binding of blood-stage antibodies on live intact sporozoites, day 15 mCherry-expressing *P. chabaudi* AS sporozoites were incubated with serum and mouse IgG was stained as above. Sporozoites were then fixed in 4% paraformaldehyde (PFA) for 15 min on ice and stained with Hoechst 33342 (1 in 1000 dilution, Molecular Probes) before being analysed on an imaging an Amnis® ImageStream®X flow cytometer at 60x magnification and low speed. Data were analysed using IDEAS® (Image Data Exploration and Analysis Software).

### 2.10 Generation of anti-*P. chabaudi* CSP polyclonal antibody and sporozoite gliding assays

A polyclonal rabbit anti- *P. chabaudi* CSP (circumsporozoite protein) was generated by Cambridge Research Biochemicals (CRB), using a peptide containing two repeat regions of PCHAS_0404100 with an N-terminal cysteine and a C-terminal amide cap: [C]-DQGGQGVQG-DQGGQGVQG-amide. Rabbits received 8 immunisations at fortnightly intervals. Rabbit antisera were affinity purified with the immunising peptide and eluted with Triethylamine (TEA). For gliding assays, live *P. chabaudi* sporozoites in 3% BSA in RPMI medium (Gibco™) were incubated with no serum, naïve sera or blood-stage sera (day 50; both sera at 1 in 100 dilution) for 30 min at 37 °C. Sporozoites were centrifuged onto glass coverslips (coated with 3% BSA in PBS) and allowed to glide for 1 h at 37 °C (5% CO_2_). Sporozoites were fixed with 4% PFA for 15 min at room temperature and blocked with 1% BSA in PBS for 1 h at room temperature. Sporozoites were incubated with anti- *P. chabaudi* CSP at 0.7ug/mL (1% BSA in PBS) for 1 h at room temperature. Sporozoites were stained with an Alexa Fluor™ 488 Anti-rabbit IgG (1 in 500 dilution, Biolegend®) for 1 h at room temperature. Cells were imaged on a Zeiss Axioimager fluorescence microscope using the 63X oil objective.

### 2.11 Statistical analysis

GraphPad Prism software version 6 or above were used for data analysis and generation of plots. For liver parasite burdens and antibody data, a non-parametric Mann-Whitney *U* test was carried out for comparison between two groups. For more than two groups a Kruskal–Wallis test with Dunn's multiple comparisons tests was carried out. A p-value of <0.05 was considered statistically significant. * = p < 0.05, ** = p < 0.01 and *** = p < 0.001. For parasitaemia data means and 95% confidence intervals were calculated on log transformed data.

## 3 Results

### 3.1 Exposure to long term *P. chabaudi* blood-stage infection cleared naturally or by chloroquine elicits partial immunity to pre-erythrocytic stages

**Fig. 1:**
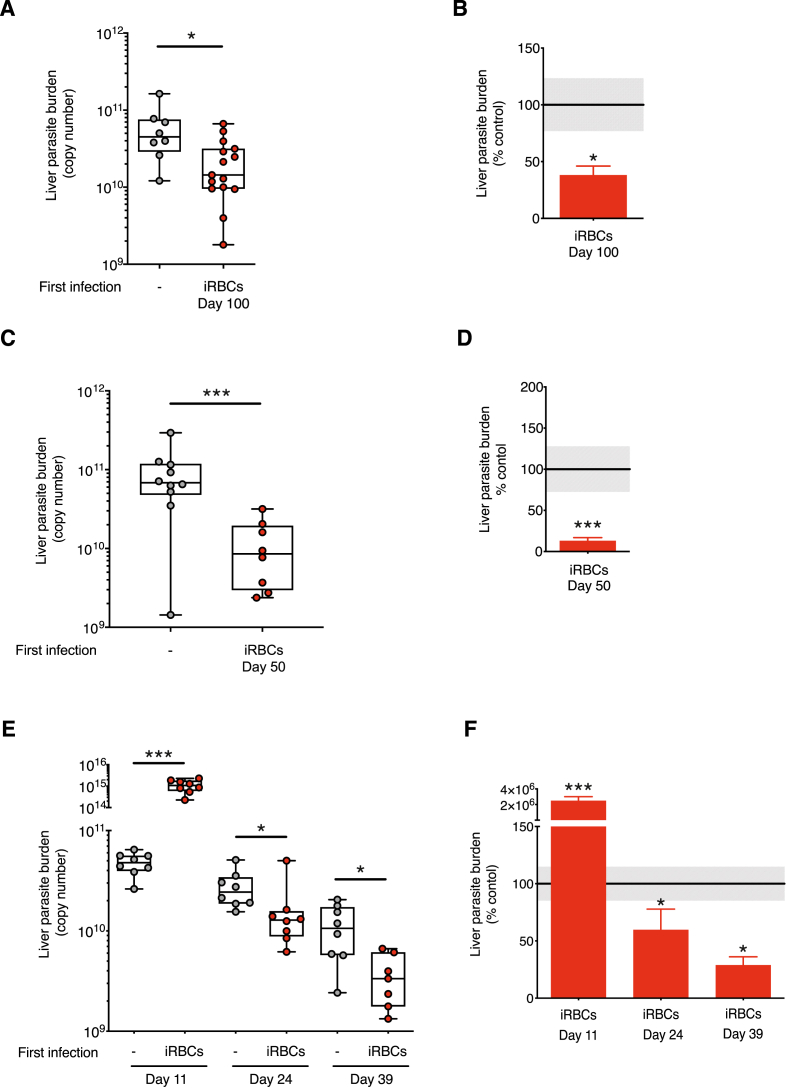
**Kinetic analysis reveals isolated exposure to *P. chabaudi* blood stages elicits protection against subsequent pre-erythrocytic stage challenge given after the peak of parasitaemia**. **A-B)** C57BL/6J mice received a first *P. chabaudi* infection with 10^5^ infected red blood cells (iRBCs) via an intraperitoneal (i.p) injection. Mice were challenged 98 days post-infection with *P. chabaudi* sporozoites via infected mosquito bites. Parasitaemia had self-cleared before sporozoite challenge. Age-matched naïve controls (−) received the sporozoite challenge only. 40–42 h post sporozoite challenge, perfused livers were harvested and processed to quantify *P. chabaudi* parasite-specific 18s rRNA by qPCR. A) Liver parasite burden presented as 18s copy number per whole liver (Liver parasite burden (copy number), individual mice shown, controls n = 8 and iRBCs Day 100 n = 15) and B) the mean percentage of the iRBCs Day 100 group relative to the control group (% control), mean + SEM. Grey shaded area represents ±SEM for the control group. **C-D)** C57BL/6J mice received a first *P. chabaudi* infection with 10^5^ iRBCs via an i.p injection. All mice were treated with chloroquine on days 24–28 to eliminate the blood-stage infection. Mice were challenged with 100 *P. chabaudi* sporozoites via an intravenous (i.v) injection 48 days after the first infection. Age-matched naïve controls (−) received the sporozoite challenge only. 40–42 h post sporozoite challenge, perfused livers were harvested and processed to quantify *P. chabaudi* parasite-specific 18s rRNA by qPCR. C) Liver parasite burden presented as 18s copy number per whole liver (Liver parasite burden (copy number), individual mice shown, controls n = 10 and iRBCs Day 50 n = 8) and D) the mean percentage of the iRBCs Day 50 relative to the control group (% control) mean + SEM. Grey shaded area represents ±SEM for the control group. **E-F)** C57BL/6J mice received a first *P. chabaudi* infection with 10^5^ iRBCs via an i.p injection. During the ongoing infection, groups of infected mice were challenged with 100 *P. chabaudi* sporozoites via an i.v injection on either day 9, 22, or 37 post-infection. At each timepoint age-matched naïve controls (−) received the sporozoite challenge only. 40–42 h post sporozoite challenge, perfused livers were harvested and processed to quantify *P. chabaudi* parasite-specific 18s rRNA by qPCR. E) Liver parasite burden presented as 18s copy number per whole liver (Liver parasite burden (copy number), individual mice shown, at each timepoint, controls n = 8 and iRBCs infected groups n = 8) and F) the mean percentage of the iRBCs infected groups relative to their corresponding control group (% control) mean + SEM. Data representative of two experiments. Grey shaded area represents ±SEM for the control group. Mann-Whitney tests were performed on liver parasite burden data for each time point, p-value *p ≤ 0.05, **p ≤ 0.01, ***p ≤ 0.001. (For interpretation of the references to colour in this figure legend, the reader is referred to the Web version of this article.)

Wildtype C57BL/6J mice were given a first infection of 10^5^ *P. chabaudi* AS iRBCs, experiencing only the blood stages of malaria infection. At 100 days post blood-stage infection after naturally eliminating their infection, mice were challenged with *P. chabaudi* AS sporozoites (delivered via mosquito bites). Mice that had only experienced blood-stage parasites, had a significantly reduced liver parasite burden compared to control mice which had not received a primary blood-stage infection (Fig. 1A), in agreement with our previous study (Nahrendorf et al., 2015). Parasite numbers in the liver at 42 h were reduced by an average of 62% with respect to a control sporozoite infection in naïve mice (Fig. 1B), confirming the induction of partial cross-stage immunity against pre-erythrocytic stages induced by the blood stages of *P. chabaudi.*

In order to determine whether the interval between primary iRBC infection and sporozoite challenge could be shortened, we infected mice with iRBCs, then at 24–28 days treated with chloroquine to remove residual blood stage parasites, and then challenged with sporozoites at day 50 days post iRBC infection. Mice immunised under this protocol also exhibited lower liver parasite burdens than naive mice infected only with sporozoites (mean percentage reduction 87.2%, (Fig. 1C and D). Subsequent experiments were conducted using either of these time intervals.

### 3.2 Cross-stage immunity to *P. chabaudi* AS is abrogated by the presence of a high blood-stage parasitaemia

In the experiments above there were no residual blood-stage parasites in the mice at the time of sporozoite challenge. As previous work has suggested that an ongoing blood-stage infection can inhibit the development of pre-erythrocytic stage immunity (Orjih and Nussenzweig, 1979; Ocana-Morgner et al., 2003), we therefore asked whether an ongoing blood-stage parasitaemia would affect the partial cross-stage protection we observed. To address this, mice were infected with iRBCs and challenged with sporozoites during the primary infection at day 9, 22 or 37, when parasites were still present in the blood.

Mice challenged with sporozoites at the peak of a blood-stage infection (day 9) were not protected (Fig. 1E). In fact, they harboured significantly greater numbers of liver-stage parasites than those seen after sporozoite infection of control naive mice, with a 2 × 10^4^ fold increase in parasite 18S rRNA. However, after the peak of the primary blood-stage infection, on days 22 and 37, when mice exhibited only low parasitaemia, partial protection against sporozoite challenge was observed (mean percentage reduction in *P. chabaudi* 18s rRNA expression of 40.3% and 71.3% at day 24 and day 39 respectively). At each timepoint the levels of *P. chabaudi* 18s rRNA expression (liver parasite burden) were also measured in a group of mice that received blood stages only and no sporozoite challenge (Sup Fig. 1). These levels of contaminating/sequestered iRBCs did not abrogate cross-stage protection after the peak of parasitaemia. Therefore, low blood-stage parasitaemia, but not acute-stage infections of *P. chabaudi* allow the development of cross-stage immunity against pre-erythrocytic stage parasites.

### 3.3 Cross-stage immunity can be induced by blood-stage *P. chabaudi* parasites derived from mosquito transmission or serially blood passaged parasites in mice

**Fig. 2:**
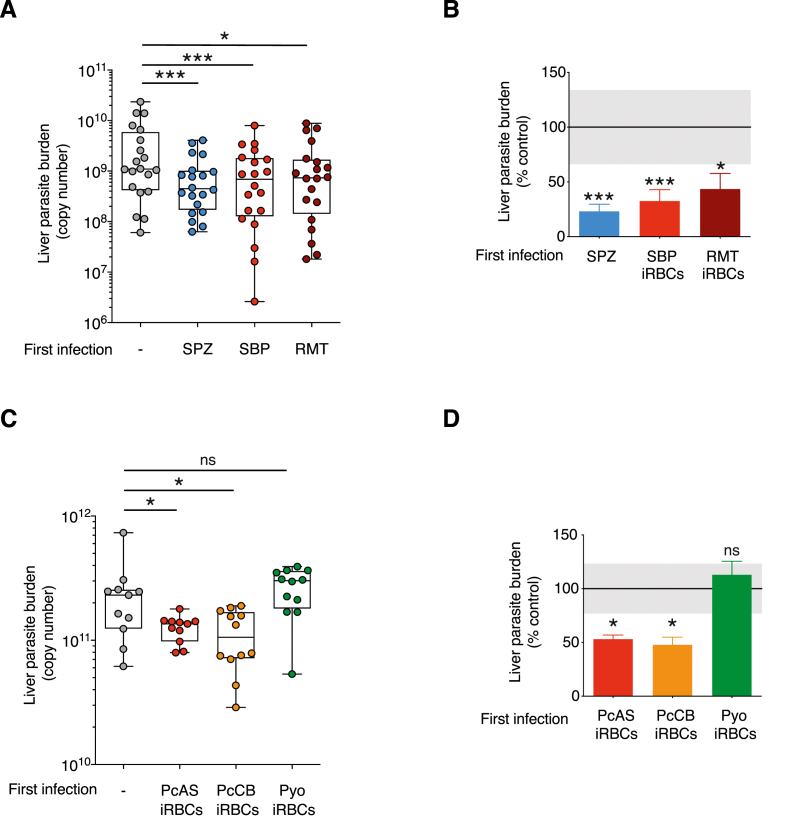
**Isolated exposure to *P. chabaudi* blood stages from different sources and strains elicit protection against sporozoite challenge**. **A-B)** C57BL/6J mice received a first *P. chabaudi* infection with either *P. chabaudi* sporozoites via infected mosquito bites (SPZ), or 10^5^ infected red blood cells (iRBCs) via an intraperitoneal (i.p) injection, derived from serial blood passage (SBP) or a recent mosquito transmission (RMT). Mice were challenged 98 days post-infection with *P. chabaudi* sporozoites via infected mosquito bites. Parasitaemia had self-cleared before sporozoite challenge. Age-matched naïve controls (−) received the sporozoite challenge only. 40–42 h post sporozoite challenge, perfused livers were harvested and processed to quantify *P. chabaudi* parasite-specific 18s rRNA by qPCR. A) Liver parasite burden presented as 18s copy number per whole liver (Liver parasite burden (copy number), individual mice shown, n = 20 per group) and B) the mean percentage of the infected groups relative to their corresponding control group (% control) mean + SEM. Grey shaded area represents ±SEM for the control group. **C-D)** C57BL/6J mice received a first blood-stage *Plasmodium* infection with either *P. chabaudi* AS (PcAS iRBCs), *P. chabaudi* CB (PcAS iRBCs), *P. yoelii* (Pyo iRBCs,) 10^5^ iRBCs via an i.p injection. All mice were treated with chloroquine on days 24–28 to eliminate the blood-stage infection. Mice were challenged with 100 *P. chabaudi* AS sporozoites via an intravenous (i.v) injection 48 days after the first infection. Age-matched naïve controls (−) received the sporozoite challenge only. 40–42 h post sporozoite challenge, perfused livers were harvested and processed to quantify *P. chabaudi* parasite-specific 18s rRNA by qPCR. C) Liver parasite burden presented as 18s copy number per whole liver (Liver parasite burden (copy number), individual mice shown, n = 11–12 per group and D) the mean percentage of the iRBCs infected groups relative to their corresponding control group (% control) mean + SEM. Data representative of two experiments. Grey shaded area represents ±SEM for the control group. Kruskal Wallis with Dunn's multiple comparisons test were performed on liver parasite burden data to determine p-values, *p ≤ 0.05, **p ≤ 0.01, ***p ≤ 0.001. (For interpretation of the references to colour in this figure legend, the reader is referred to the Web version of this article.)

We have previously established that *P. chabaudi* blood-stage parasites derived from recently mosquito-transmitted infection (RMT) differ from serially blood-passaged (SBP) iRBCs in the magnitude of the blood-stage infection, as well in virulence and parasite transcriptome (Spence et al., 2013, Brugat et al., 2017b) Sup Fig. 2). We therefore asked whether the cross-stage protection afforded by RMT *P. chabaudi* AS, as described in Nahrendorf et al (2015) and above (Fig. 1A), was a consequence of antigens expressed only in RMT blood stages, or whether protection could also be achieved using SBP parasites (Fig. 2A). Mice infected with SBP blood stages had a significantly lower number of liver-stage parasites upon sporozoite challenge (76% reduction in liver-stage parasites compared with sporozoite infection controls), similar to that achieved with mice infected with RMT iRBCs (62% reduction, Fig. 2B). As expected, mice previously infected with sporozoites, thus experiencing all pre-erythrocytic stages and blood stages, were partially protected upon sporozoite challenge 100 days after initial infection. We conclude that the antigens responsible for cross-stage immunity to sporozoites induced by blood stages are present on both serially blood-passaged parasites, and those which have recently been transmitted through mosquitoes.

### 3.4 Cross-stage immunity is observed between *P. chabaudi* strains, but not against another rodent *Plasmodium* species

To investigate the species and strain specificity of cross-stage immunity to *P. chabaudi* pre-erythrocytic stages, mice were given a blood-stage infection of *P. chabaudi* AS, a more virulent strain *P. chabaudi* CB or a different species, *P. yoelii* 17XL. As above, *P. chabaudi* AS blood stages partially protected against homologous challenge with *P. chabaudi* AS sporozoites (47% reduction in liver stage parasites, Fig. 2C and D). Similarly, *P. chabaudi* CB strain partially protected against heterologous challenge with *P. chabaudi* AS sporozoites with a mean reduction in liver-stage parasites of 52%. A previous infection with *P. yoelii* 17XL blood stages, however, did not result in a significant reduction in liver parasite burden upon challenge with *P. chabaudi* AS sporozoites.

### 3.5 Cross-stage immunity to *P. chabaudi* sporozoite challenge is not mediated by effector CD4^+^ or CD8^+^ T cells

**Fig. 3:**
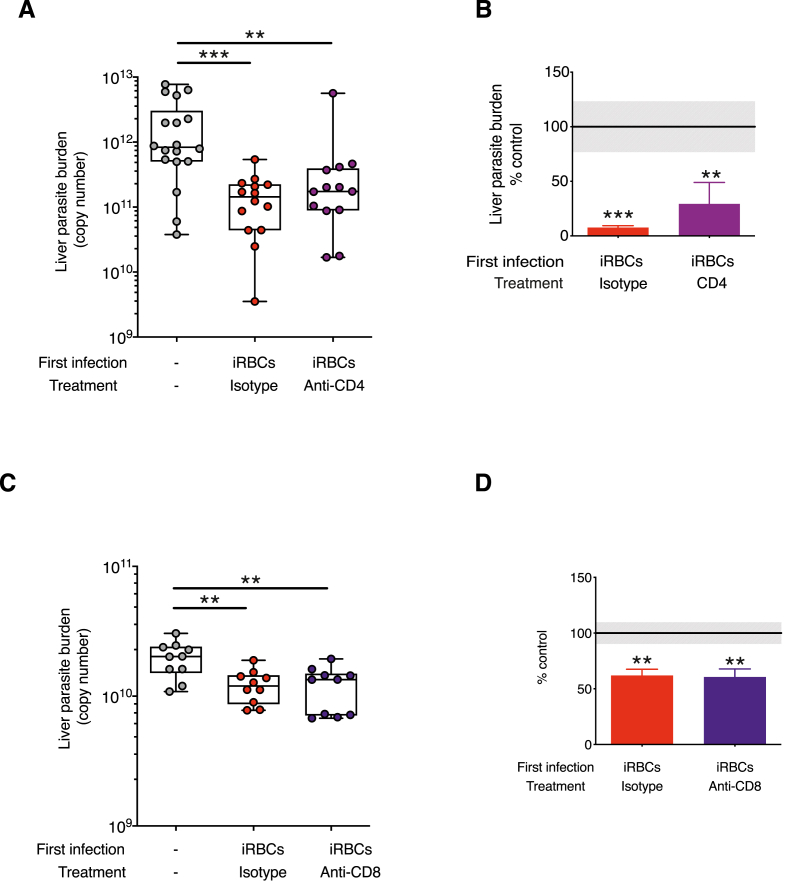
**CD8**^**+**^**and CD4 T**^**+**^**cells are not required for cross-stage immunity to *P. chabaudi* pre-erythrocytic challenge infection. A-B)** C57BL/6J mice received a first *P. chabaudi* infection with 10^5^ infected red blood cells (iRBCs) via an intraperitoneal (i.p) injection. On days 94–99 mice received treatment with either an anti-CD4 depleting antibody (clone YTS 191) or control isotype rat IgG antibodies. Mice were challenged 98 days post-infection with *P. chabaudi* sporozoites via infected mosquito bites. Parasitaemia had self-cleared before sporozoite challenge. Age-matched naïve controls (−) received the sporozoite challenge only. 40–42 h post sporozoite challenge, perfused livers were harvested and processed to quantify *P. chabaudi* parasite-specific 18s rRNA by qPCR. A) Liver parasite burden presented as 18s copy number per whole liver (Liver parasite burden (copy number), individual mice shown, n = 13–18 per group) and B) the mean percentage of the iRBCs infected groups relative to the corresponding control group (% control) mean + SEM. Grey shaded area represents ±SEM for the control group. **C-D)** C57BL/6J mice received a first *P. chabaudi* infection with 10^5^ iRBCs via an i.p injection. All mice were treated with chloroquine on days 24–28 to eliminate the blood-stage infection. On days 44–49 mice received either anti-CD8β (Lyt 3.2, clone 53-5.8) depleting or isotype control antibodies. Mice were challenged with 100 *P. chabaudi* sporozoites via an intravenous (i.v) injection 48 days after the first infection. Age-matched naïve controls (−) received the sporozoite challenge only. 40–42 h post sporozoite challenge, perfused livers were harvested and processed to quantify *P. chabaudi* parasite-specific 18s rRNA by qPCR. C) Liver parasite burden presented as 18s copy number per whole liver (Liver parasite burden (copy number), individual mice shown, n = 10 per group) and D) the mean percentage of the iRBCs infected groups relative to the control group (% control) mean + SEM. Data representative of two experiments. Grey shaded area represents ±SEM for the control group. Kruskal Wallis with Dunn's multiple comparisons test were performed on liver parasite burden data to determine p-values, *p ≤ 0.05, **p ≤ 0.01, ***p ≤ 0.001. (For interpretation of the references to colour in this figure legend, the reader is referred to the Web version of this article.)

The mechanisms mediating cross-stage immunity in *P. chabaudi* are unknown, but it is commonly believed that protective immunity against pre-erythrocytic liver stages of other *Plasmodium* species is primarily mediated by T cells (Weiss et al., 1988; Tsuji and Zavala, 2003). To investigate the contribution of effector T cells in mediating control of exoerythrocytic stages of *P. chabaudi*, antibodies to CD4^+^ or CD8^+^ T cells (anti-CD8β) or isotype control antibodies were administered to mice which had previously eliminated a *P. chabaudi* blood-stage infection, 3 days and 1 day prior to, and 1 day after sporozoite challenge (Fig. 3). Mice that received the depleting antibodies had fewer than 1% CD4^+^ or CD8^+^ T cells respectively remaining in the liver and spleen (Sup Fig. 3). Depletion of CD4^+^ T cells prior to sporozoite challenge did not abrogate cross-stage protection. The depleted group had a mean percentage reduction of 70.7% and the isotype controls had a mean percentage reduction of 92.4% and there was no significant difference between these groups (Fig. 3A and B). Following CD8^+^ T cell depletion, mice had a mean percentage reduction of 39.5% compared with the primary infection. The isotype control group had a mean percentage reduction of 38.2%. There was no difference between isotype controls and treated groups (Fig. 3C and D).

### 3.6 Blood-stage infection with *P. chabaudi* induces antibodies that recognise antigens on sporozoites

**Fig. 4:**
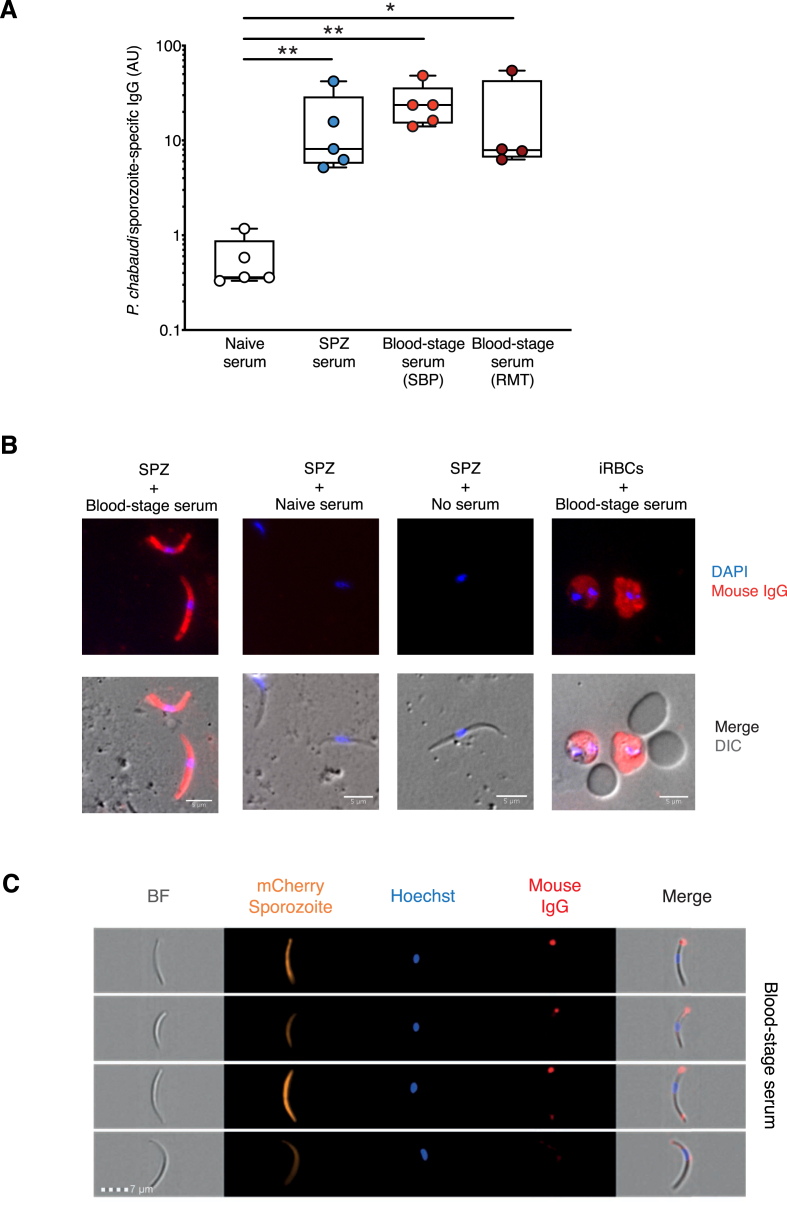
**Blood-stage *P. chabaudi* infection induces antibodies that recognise both lysed and the surface of whole sporozoites**. **A)** C57BL/6J mice received a *P. chabaudi* infection with either *P. chabaudi* sporozoites via infected mosquito bites (SPZ), or 10^5^ infected red blood cells (iRBCs) via an intraperitoneal (i.p) injection, derived from serial blood passage (SBP) or a recent mosquito transmission (RMT). Age-matched naïve mice were used as controls. On day 100 post infection blood was taken to generate serum. ELISAs were performed with these sera against a *P. chabaudi* sporozoite lysate to measure levels of sporozoite specific total mouse IgG generated after these infections. Data expressed as AU, arbitrary units as described in the materials and methods. Individual mice shown, n = 5 per group. Data representative of two duplicates. Mann-Whitney tests were performed to compare each serum against naïve controls p-value *p ≤ 0.05, **p ≤ 0.01, ***p ≤ 0.001. **B)** C57BL/6J mice were given 10^5^ iRBCs via an i.p injection, to initiate a blood-stage infection. On day 100 post infection blood was taken to generate serum. Sporozoites were fixed and permeabilised on glass slides with methanol:acetone followed by 0.5% Triton X-100 in PBS. Slides were incubated with naïve or blood-stage serum. Thin blood smears were made from a mouse day 7 post SBP iRBCs infection, fixed, permeabilised and incubated with blood-stage serum. All slides were stained with a goat anti-mouse IgG antibody and slides were mounted with a medium containing DAPI. DIC, Differential Interference Contrast. Scale bar, 5 μm. Images are representative of 50 parasites stained. **C)** C57BL/6J mice were given 10^5^*P. chabaudi* iRBCs, to initiate a blood-stage infection. On day 100 post infection blood was taken to generate serum. Live mCherry-expressing *P. chabaudi* sporozoites were incubated with serum from an MT blood-stage infection, naive serum or no serum (controls). Next, sporozoites were incubated with a goat anti-mouse IgG antibody and then fixed with 4% PFA. The reactivity of anti-mouse IgG in Hoechst positive cells was analysed via ImageStream®X imaging cytometry. BF, brightfield. Scale bar, 7 μm. Images are representative of 500 parasites stained. (For interpretation of the references to colour in this figure legend, the reader is referred to the Web version of this article.)

As cross-stage immunity to *P. chabaudi* sporozoite challenge was not mediated by effector T cells, we investigated whether B cells and/or antibodies were responsible for the protective phenotype observed. Sera taken from an RMT or SBP blood-stage infection at 100 days post infection, as well as sera derived from a mosquito-transmitted infection (SPZ serum) were tested in ELISA assays for reactivity with lysates prepared from *P. chabaudi* sporozoites. Sera from both RMT and SBP blood-stage infections contained antibodies which reacted with sporozoites at similar levels to mice infected with sporozoites (Fig. 4A). Immunofluorescence assays also confirmed that blood-stage serum IgG antibodies bind to lysed sporozoites and iRBCs (Fig. 4B).

Imaging flow cytometry confirmed that blood-stage antibodies also bind unlysed intact sporozoites (Fig. 4C), whilst naïve mouse serum and no serum controls displayed no binding (Sup Fig. 4). The binding of blood-stage antibodies suggests a surface location of the recognised antigens. In this case, staining appears to be localised to the apical ends of the sporozoites. This may reflect that the antibodies recognise antigens only at the tips of the sporozoite.

### 3.7 Blood-stage antibodies can impede sporozoite motility

**Fig. 5:**
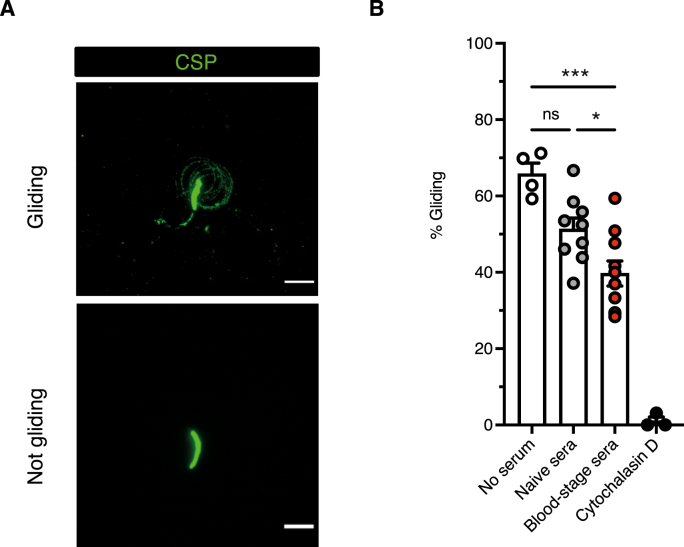
**Blood-stage antibodies impair sporozoite motility.***P. chabaudi* salivary gland sporozoites were allowed to glide on BSA-coated coverslips for 1 h at 37 °C. Coverslips were then fixed with 4% PFA. *P. chabaudi* sporozoites trails were detected using a polyclonal anti-P. chabaudi CSP antibody, followed by an anti-rabbit IgG secondary antibody. A) Representative images of a sporozoite gliding or not gliding are shown. B) Each data point represents an individual coverslip where the whole coverslip was scanned and the percentage of sporozoites gliding was enumerated. Sporozoites were pre-treated with no serum, naïve sera, blood-stage serum (serum taken day 50 post SBP iRBCs infection) or Cytochalasin D (1 μM). Sera from different mice were used for each coverslip, n = 9–10. One-way ANOVA with Tukey's multiple comparisons test was performed to determine p-values, *p ≤ 0.05, **p ≤ 0.01, ***p ≤ 0.001, ****p ≤ 0.0001.

Inhibition of the gliding motility of sporozoites *in vitro* has been used as a measure of the protective capacity of antibodies that react with the surface of *Plasmodium* sporozoites (Stewart and Vanderberg, 1988; Santos et al., 2017). Accordingly, we asked whether the antibodies generated during a *P. chabaudi* blood-stage infection affected sporozoite gliding motility. Sera from *P. chabaudi* RMT blood-stage infections (day 50 post infection) were incubated with live sporozoites and gliding was measured. Trails resulting from gliding activity were detected using an anti-*P. chabaudi* CSP peptide antiserum generated in the laboratory (Fig. 5A, Sup Fig. 5). Up to 65% of sporozoites exhibited gliding motility in medium without mouse serum (Fig. 5B). Sporozoites incubated with blood-stage sera moderately inhibited gliding motility; the percentage of gliding trails were significantly lower in the presence of blood-stage sera compared to gliding in the presence of control naïve sera (39.7% and 51.3% motility respectively). Cytochalasin D afforded 99% gliding inhibition. In summary, antibodies obtained from a blood-stage *P. chabaudi* infection are able to significantly impede sporozoite gliding, suggesting a possible mechanism for the cross-stage immunity we observe *in vivo.*

### 3.8 B cells and antibodies are required for cross-stage immunity in *P. chabaudi*

**Fig. 6:**
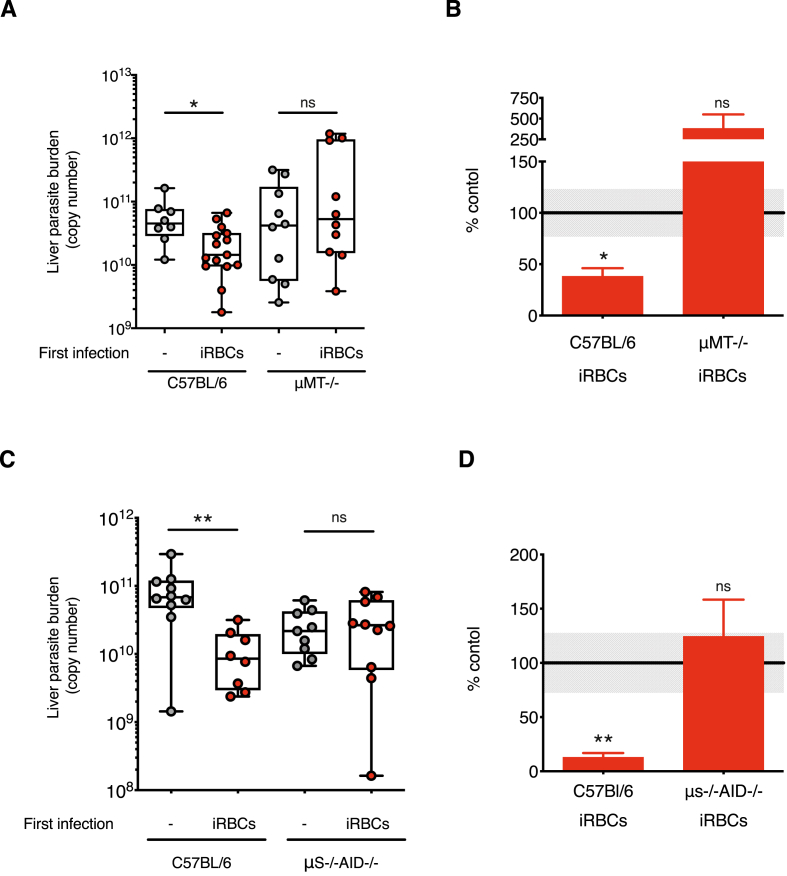
**B cells and secreted blood-stage antibodies are essential for cross-stage immunity to *P chabaudi* pre-erythrocytic stage infection. A-B)** Wildtype C57BL/6J and μMT−/− (B cell deficient) mice received a first *P. chabaudi* infection with 10^5^ infected red blood cells (iRBCs) via an intraperitoneal (i.p) injection. Mice were challenged 98 days post-infection with *P. chabaudi* sporozoites via infected mosquito bites. All mice were treated with chloroquine on days 24–28 to eliminate the blood-stage infection before sporozoite challenge. Age-matched naïve controls (−) received the sporozoite challenge only. 40–42 h post sporozoite challenge, perfused livers were harvested and processed to quantify *P. chabaudi* parasite-specific 18s rRNA by qPCR. A) Liver parasite burden presented as 18s copy number per whole liver (Liver parasite burden (copy number), individual mice shown, n = 17–24 per group) and B) the mean percentage of the iRBCs infected groups relative to the corresponding control group (% control) mean + SEM. Grey shaded area represents ±SEM for the control group. **C-D)** Wildtype C57BL/6J and μS-AID−/− (unable to secrete antibodies) mice received a first *P. chabaudi* infection with 10^5^ iRBCs via an i.p injection. All mice were treated with chloroquine on days 24–28 to eliminate the blood-stage infection. Mice were challenged with 100 *P. chabaudi* sporozoites via an intravenous (i.v) injection 48 days after the first infection. Age-matched naïve controls (−) received the sporozoite challenge only. 40–42 h post sporozoite challenge, perfused livers were harvested and processed to quantify *P. chabaudi* parasite-specific 18s rRNA by qPCR. C) Liver parasite burden presented as 18s copy number per whole liver (Liver parasite burden (copy number), individual mice shown, n = 10 per group) and D) the mean percentage of the iRBCs infected groups relative to the corresponding control group (% control) mean + SEM. Grey shaded area represents ±SEM for the control group. Data representative of two experiments. Kruskal Wallis with Dunn's multiple comparisons test were performed on liver parasite burden data to determine p-values, *p ≤ 0.05, **p ≤ 0.01, ***p ≤ 0.001. (For interpretation of the references to colour in this figure legend, the reader is referred to the Web version of this article.)

Antibodies which recognise sporozoites are clearly induced by isolated exposure to blood-stage parasites. These antibodies may impede sporozoite gliding and play a role in the cross-stage immunity we have demonstrated *in vivo*. We therefore investigated whether the humoral response induced by a blood-stage *P. chabaudi* infection was effective at reducing the numbers of exoerythrocytic *P. chabaudi* parasites *in vivo*. For this, we used μMT mice lacking B cells and antibodies (Kitamura et al., 1991) which previously demonstrated the importance of B cells in eliminating blood-stage *P. chabaudi* infections (von der Weid et al., 1996). We also employed μS−/−AID−/− mice (Kumazaki et al., 2007), which have B cells, but lack the ability to secrete antibodies in naïve and *Plasmodium* infected mice (Sup Fig. 6), allowing us to determine the effects of secreted antibodies rather than another function of B cells. Both μMT (von der Weid et al., 1996), and μS−/−AID/- mice (Sup Fig. 7), were unable to clear chronic blood-stage infections completely and therefore were given the antimalarial drug chloroquine on days 26–28 post infection to eliminate parasites before being challenged with sporozoites at day 100 or 50 respectively. The protective effect of a blood-stage *P. chabaudi* infection on liver parasites was completely abrogated in μMT and μs−/−AID−/− mice (Fig. 6A and B). In both cases, the numbers of liver-stage parasites were not significantly different from those observed in naïve mice receiving only a sporozoite challenge. In these experiments, wild-type C57BL/6J mice previously infected with blood-stage *P. chabaudi* were, as expected, partially immune to sporozoite challenge, with a 60–80% reduction in liver parasite numbers compared to controls (Fig. 6).

## 4 Discussion

A *Plasmodium* infection initiated by the natural route of injection of sporozoites leads to the development of both pre-erythrocytic and blood stages of the parasite in the mammalian host. Upon a secondary challenge infection with sporozoites, it is therefore difficult to determine the contribution of stage-specific and cross-stage immune responses to protective immunity. It would be important to understand whether cross-stage immunity plays a significant role in immunity to malaria, as antigens shared by different parasite stages could be useful additions to a malaria vaccine. Here we utilised *P. chabaudi* parasites in mice to explore the protective effect of one stage of the parasite life cycle in the mouse, upon challenge with another life-cycle stage. We found that a blood-stage infection initiated directly with infected RBCs gives partial protection against a sporozoite challenge resulting in a significant reduction in liver parasite burdens. This cross-stage immunity is parasite strain-transcending and antibody-dependent.

Our study using *P. chabaudi* in C57BL/6J mice shows significant cross-stage immunity after a single preceding blood-stage infection. There are few reports specifically addressing cross-stage immunity in experimental models of malaria; however, some immunity to *P. yoelii* sporozoite challenge is generated in mice that had a *P. yoelii* blood-stage infection, or after immunisation with a *P. yoelii* blood-stage parasite lysate (Belnoue et al., 2008; Lu et al., 2017). In our case using *P. chabaudi* parasites, pre-erythrocytic stage immunity was observed in mice challenged 50–100 days after a blood-stage infection, after patent blood-stage parasitaemia was cleared. This partial immunity was effective against a different strain of *P. chabaudi* (CB) which is known to be more virulent (Cheesman et al., 2006). We have shown that during a blood-stage infection and after mosquito transmission, these parasite strains are transcriptionally and phenotypically different (Lin et al., 2018). Strain specific immunity has been reported between *P. chabaudi* AS and *P. chabaudi* CB blood stages (Jarra and Brown, 1985), however despite their differences, here we demonstrate *P. chabaudi* CB blood stages can elicit cross-protection to AS strain sporozoites, suggesting antigens conserved across strains are able to induce cross-stage immunity. The ability to mount strain-transcending immunity to malaria would be an attractive characteristic for a potential malaria vaccine.

Immunisation with blood-stage parasites from a different species of rodent malaria, *P. yoelii,* did not yield protection against subsequent *P. chabaudi* AS sporozoite challenge. These results mirror a report that after immunisation with *P. berghei* ANKA iRBCs under chloroquine cover mice are not protected against *P. yoelli* sporozoite challenge (Belnoue et al., 2008). Experimental models are required to elucidate pre-erythrocytic stage mechanisms of protection and differences in liver parasite burdens. Thus, the specificity of cross-stage immunity in *P. chabaudi* investigated here is an important consideration as malaria-endemic regions often have multiple species in circulation and genetic diversity has been reported in different isolates of the same species (Mayxay et al., 2004; Meyer et al., 2002). Therefore, identification of cross-stage antigens shared between different parasite strains would be of value for malaria vaccines inducing strain-transcending immunity.

Challenge with sporozoites during an ongoing *P. chabaudi* blood-stage infection had different outcomes depending on the time of challenge. Administration of sporozoites during the peak of acute blood-stage infection resulted in enhanced rather than reduced liver parasite burdens, whilst mice were partially protected when challenged after the peak, when parasitaemias were lower. A previous study suggested that a concurrent blood-stage infection may inhibit immunity to pre-erythrocytic stages of *P. berghei* stages (Orjih and Nussenzweig, 1979). Similarly, blood-stage induced dendritic cells have been shown to suppress liver CD8^+^ T cell responses (Ocana-Morgner et al., 2003). By contrast, another study reports that superinfection of sporozoites on a blood-stage infection reduces liver-stage parasites (Portugal et al., 2011). The differences between the studies may well be dependent on the magnitude of blood parasitaemia and the exact timing, which were not investigated. The reduction of pre-erythrocytic *P. chabaudi* parasites described here is more in line with the experiments of Portugal et al. However, their study suggests that the reduction of liver-stage parasites is dependent on hepcidin, and not specific immunity. In our case, the species specificity of cross-stage immunity with *P. chabaudi* infections would strongly suggest that immune mechanisms are involved.

We measured liver parasite burden as an indication of cross-stage protective immunity induced by blood-stage infection; however, this data alone cannot distinguish whether the reductions we see are due to a host mechanism preventing invasion of hepatocytes and/or immune removal of infected cells in the liver. There is a large body of work implicating CD8^+^ T cells as the main effectors of liver-stage immunity with cytotoxic cells killing infected hepatocytes, and liver-tissue resident memory (Trm) CD8^+^ T cells specifically being essential for liver stage immunity, in the context of sporozoite vaccination (Weiss et al., 1988; Tsuji and Zavala, 2003; Fernandez-Ruiz et al., 2016).

Here, however, depletion of either CD8^+^ T cells or CD4^+^ T cells prior to *P. chabaudi* sporozoite challenge did not abrogate the protection observed. It is possible that a single *P. chabaudi* blood-stage infection may not produce sufficient CD8^+^ T cells or liver-resident CD8^+^ T cells that could mediate this cross-stage immunity. Our results contrast with previous studies using higher numbers of *P. yoelii* or large amounts of parasite lysate where depletion of CD8^+^ T cells did abrogate cross-stage protection to sporozoite challenge (Belnoue et al., 2008; Lu et al., 2017). In *Plasmodium* immunity, the threshold of CD8^+^ T cells required is thought to be very high in comparison to the response required to clear bacterial or viral infections (Schmidt et al., 2008). This together with the report that generation of CD8^+^ T cell responses to pre-erythrocytic stage parasites is defined by the initial antigen dose and subsequent antigen exposure does not affect the magnitude of these responses (Hafalla et al., 2002), may partially explain the differences in the studies. Increasing the infecting dose or use of *P. chabaudi* strains which have higher parasitaemias may well affect the contribution of CD8^+^ T cells in this cross-stage immunity, and thus increase the protective efficacy.

A single *P. chabaudi* blood-stage infection elicits antibodies that react with the surface of sporozoites, and they appear to bind the apical ends of sporozoites. This suggests that they may have some functional importance in protective immunity. Proteins secreted and localised to the apical ends of liver infective sporozoites such as CSP, AMA-1 (apical merozoite antigen 1) and some rhoptry neck proteins, have been shown to be essential for complete parasite invasion (Stewart and Vanderberg, 1991; Tokunaga et al., 2019; Yang et al., 2017; Arredondo et al., 2021).

There is a large body of evidence demonstrating that antibody responses against sporozoites are protective (Flores-Garcia et al., 2018; Arevalo-Herrera et al., 2016; Vanderberg, 2015; Dups et al., 2014). Antibodies produced following a sporozoite infection can inhibit the release of sporozoites from the mosquito proboscis into the skin in the case of a subsequent infection (Kebaier et al., 2009), neutralise sporozoites directly, or by opsonisation, thereby preventing hepatic invasion (Nussenzweig et al., 1972) or inhibit liver-stage development (Chatterjee et al., 1999). In particular, anti-sporozoite and anti-CSP antibodies have influenced malaria vaccine development, notably the RTS,S vaccine (Spitalny and Nussenzweig, 1973; Milich et al., 2001; Chappel et al., 2004; White et al., 2015).

Apicomplexan parasites such as *Plasmodium* sporozoites move in a substrate-dependent manner known as gliding motility, and this movement is required for migration to the liver for invasion of hepatocytes *in vivo* and can be visualised *in vitro* by tracking CSP trails (Frenal et al., 2017; Vanderberg, 1974). Sera obtained from mice that have recovered from a single blood-stage *P. chabaudi* infection can inhibit the gliding motility of sporozoites *in vitro*. Gliding inhibition assays have previously been shown to reflect the protective capacity of anti-sporozoite antibodies (Stewart et al., 1986), although protective antibodies have been described that do not prevent gliding motility (Tan et al., 2021).

B cells and antibodies have previously been shown to be critical for the clearance of *P. chabaudi* blood-stage parasites (von der Weid et al., 1996; Langhorne et al., 1998; Perez-Mazliah et al., 2015). We show here that they also contribute to cross-stage immunity to pre-erythrocytic stages elicited by the blood-stage infection, as it was not possible to protect previously blood-stage-infected mice lacking mature B cells and/or secreted antibodies from a sporozoite challenge. In mice, multiple *P. chabaudi* blood-stage infections leads to the generation of hyperimmune serum that protects against homologous challenge infection (Jarra et al., 1986). Increasing the levels of cross-stage antibodies could improve protection. It is also important to note that within immune sera there may be interference from non-neutralising antibodies which may hinder the protective capacity of cross-stage antibodies (Vijayan et al., 2021). Future work could investigate whether increasing cross-stage antibody levels could enhance *P. chabaudi* cross-stage immunity.

The contribution of cross-stage antibody responses elucidated here adds an additional layer to our understanding of how individuals can be protected against *Plasmodium* infection. It would be of great interest for future studies to characterise and identify the antigenic targets of the cross-stage antibodies within the polyclonal blood-stage serum. Identification of these protective strain-transcending cross-stage antigens will aid advancements in malaria vaccine development.

## Funding

This work was supported by the 10.13039/100010438Francis Crick Institute which receives its core funding from the UK Medical Research Council (FC001101), 10.13039/501100000289Cancer Research UK (FC001101) and the 10.13039/100010269Wellcome Trust (FC001101); JL is a 10.13039/100010269Wellcome Trust Senior Investigator (grant reference WT101777MA). ITD is the recipient of a Francis Crick PhD studentship.

## CRediT authorship contribution statement

**Irene Tumwine-Downey:** Conceptualization, Methodology, Investigation, performed experiments and, Formal analysis, Visualization, Writing – original draft, Writing – review & editing. **Katrien Deroost:** Conceptualization, Methodology, microscopy and flow cytometry, Investigation, serum and liver harvesting, Writing – review & editing. **Prisca Levy:** Methodology, gliding assay, Investigation, mosquito breeding and transmissions, mosquito dissections and infections, serum and liver harvesting, Writing – review & editing. **Sarah McLaughlin:** Investigation, liver harvesting, mouse breeding and genotyping, Writing – review & editing. **Caroline Hosking:** Methodology, ELISA protocols and, Formal analysis, Investigation, mosquito dissections and infections, liver harvesting, Writing – review & editing. **Jean Langhorne:** Conceptualization, Methodology, Writing – original draft, Writing – review & editing, Supervision, Funding acquisition.

## Declaration of competing interest

The authors declare the following financial interests/personal relationships which may be considered as potential competing interests:Jean Langhorne on the Editorial Board of Current Research in Immunology (JL).

## Data Availability

Data will be made available on request.
