# Supplementary material for: Antibody-dependent immune responses elicited by blood stage-malaria infection contribute to protective immunity to the pre-erythrocytic stages
Source: Curr Res Immunol. 2022 Dec 23;4:100054. doi: 10.1016/j.crimmu.2022.100054 (PMC9803926; doi:10.1016/j.crimmu.2022.100054)

SUP FIGURE 1

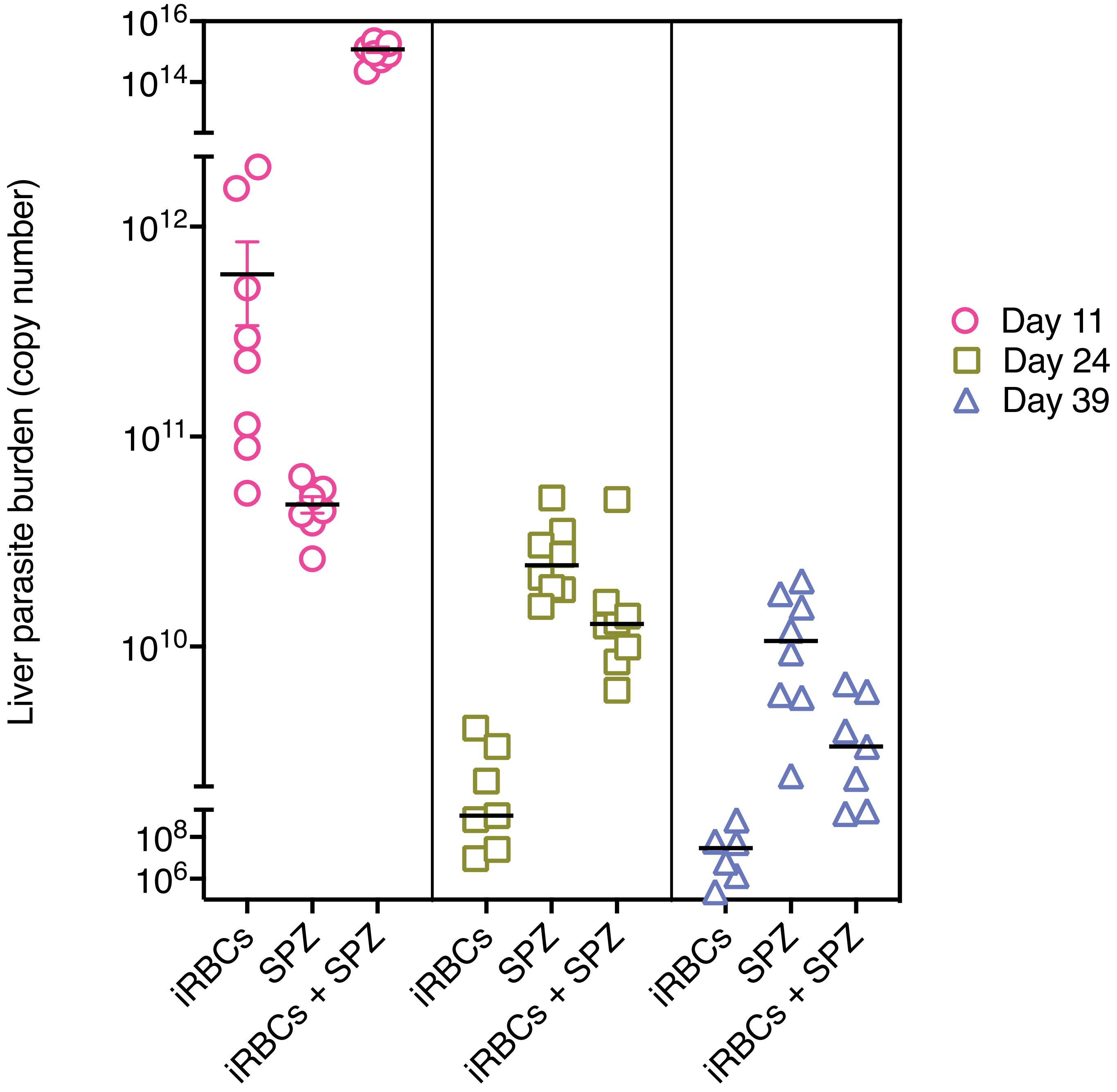
Liver parasite burden (copy number)
10^{16}
10^{14}
10^{12}
10^{11}
10^{10}
10^{8}
10^{6}
iRBCs
SPZ
iRBCs + SPZ
iRBCs
SPZ
iRBCs + SPZ
iRBCs
SPZ
iRBCs + SPZ
Day 11
Day 24
Day 39

SUP FIGURE 2

A RMT infection

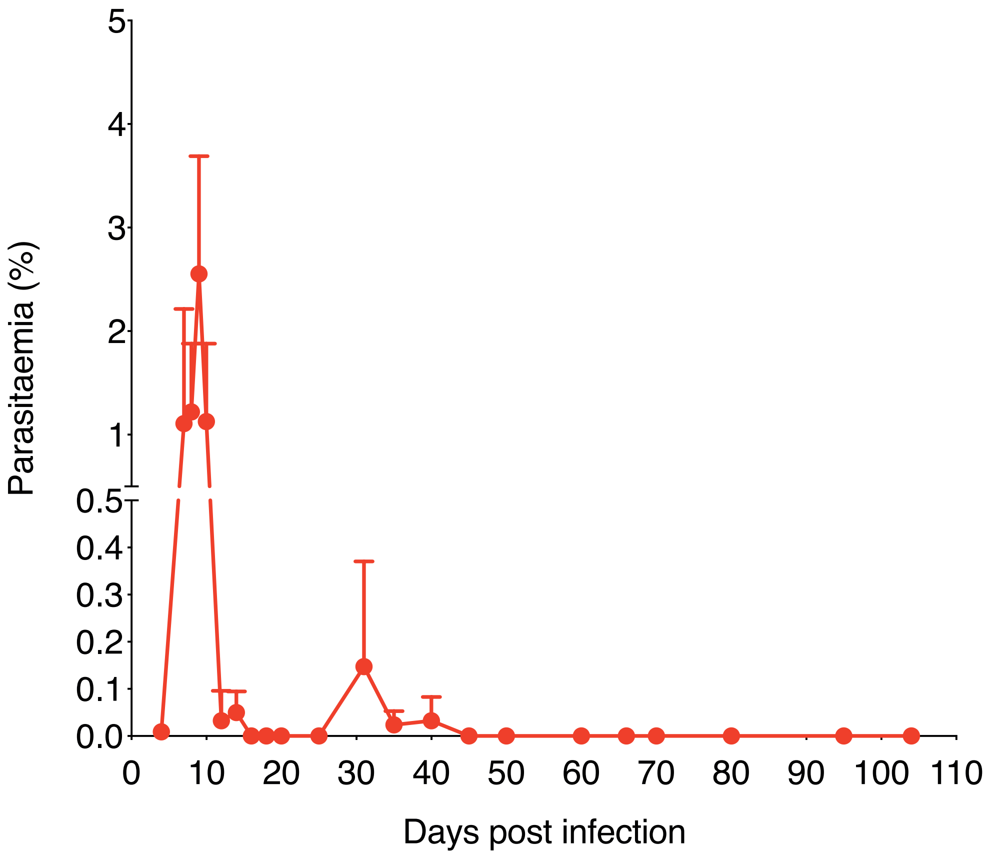

B SBP infection

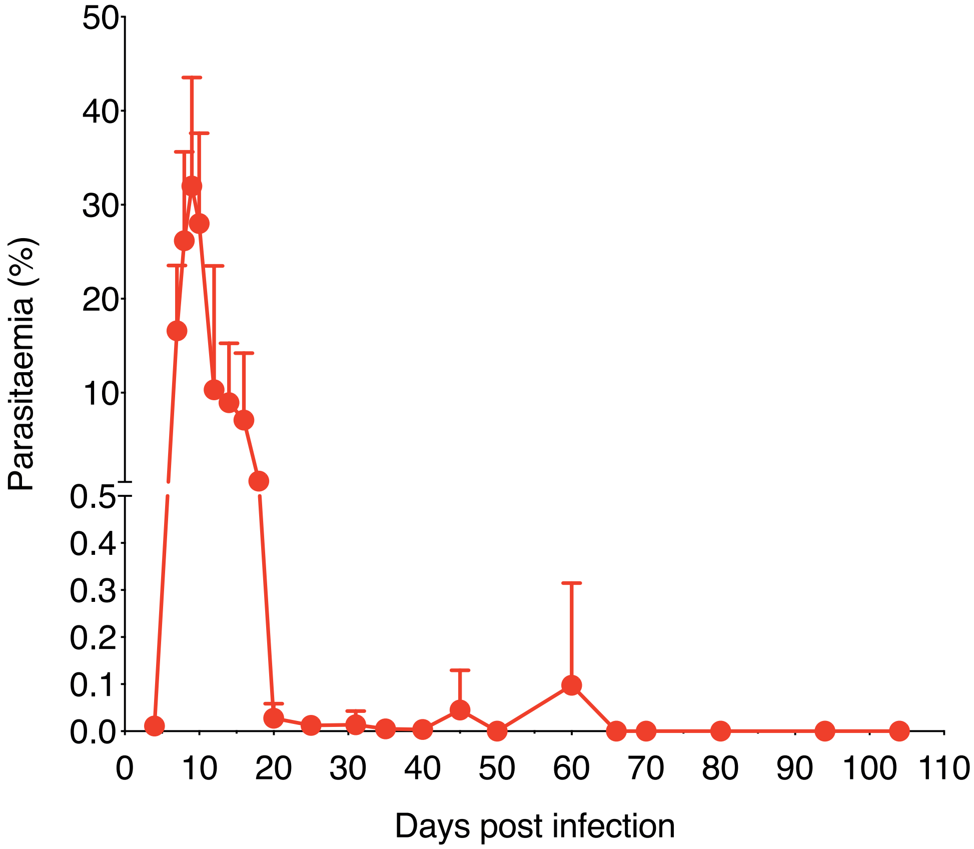

SUP FIGURE 3

A

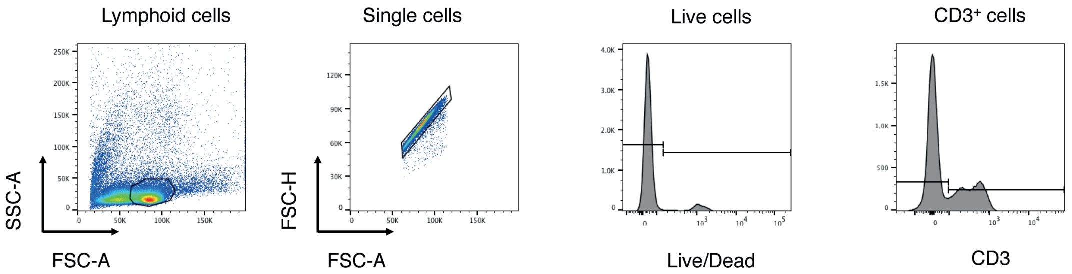

B

Gated on single, live CD3+ lymphocytes

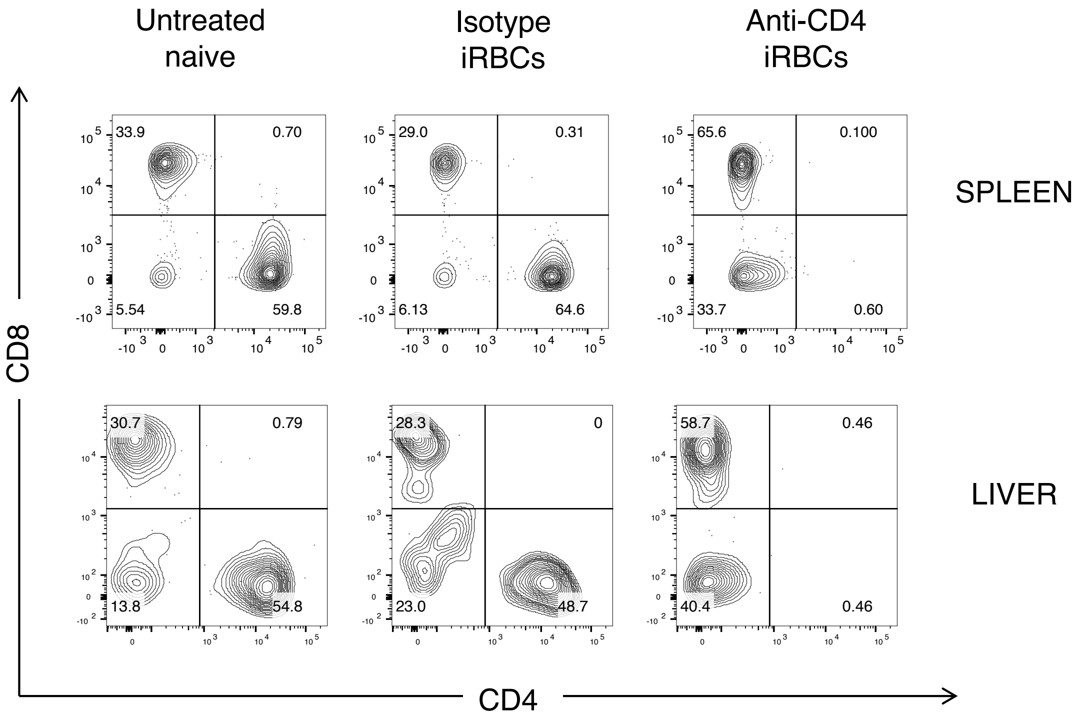

C

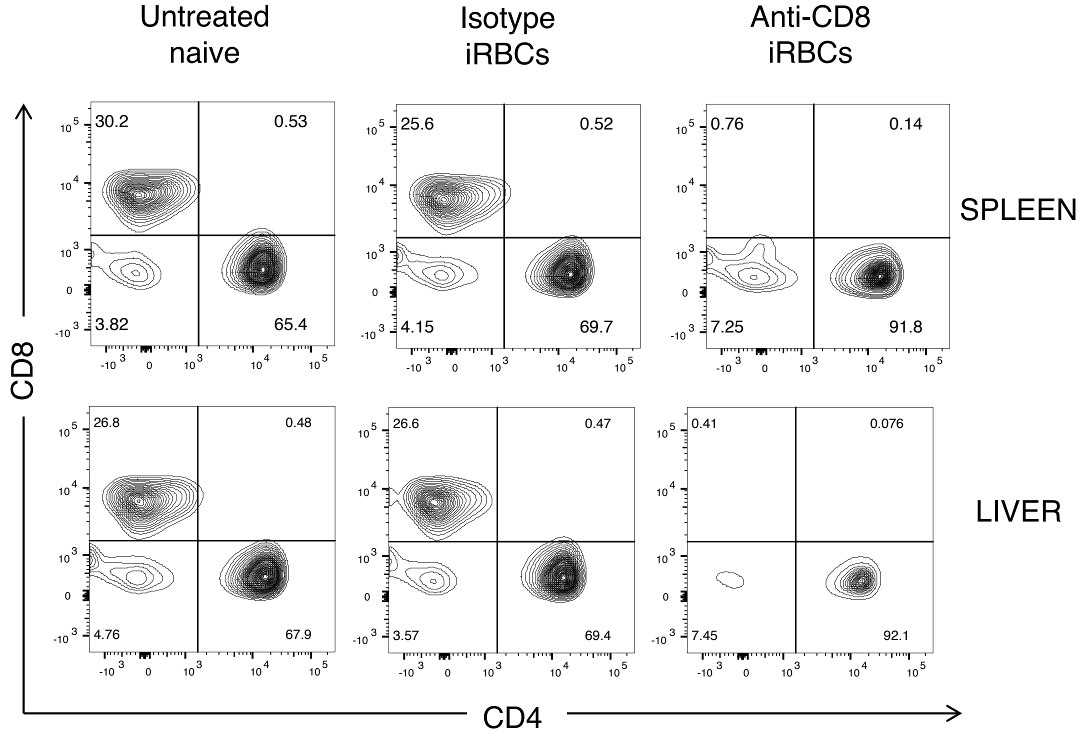

SUP FIGURE 4

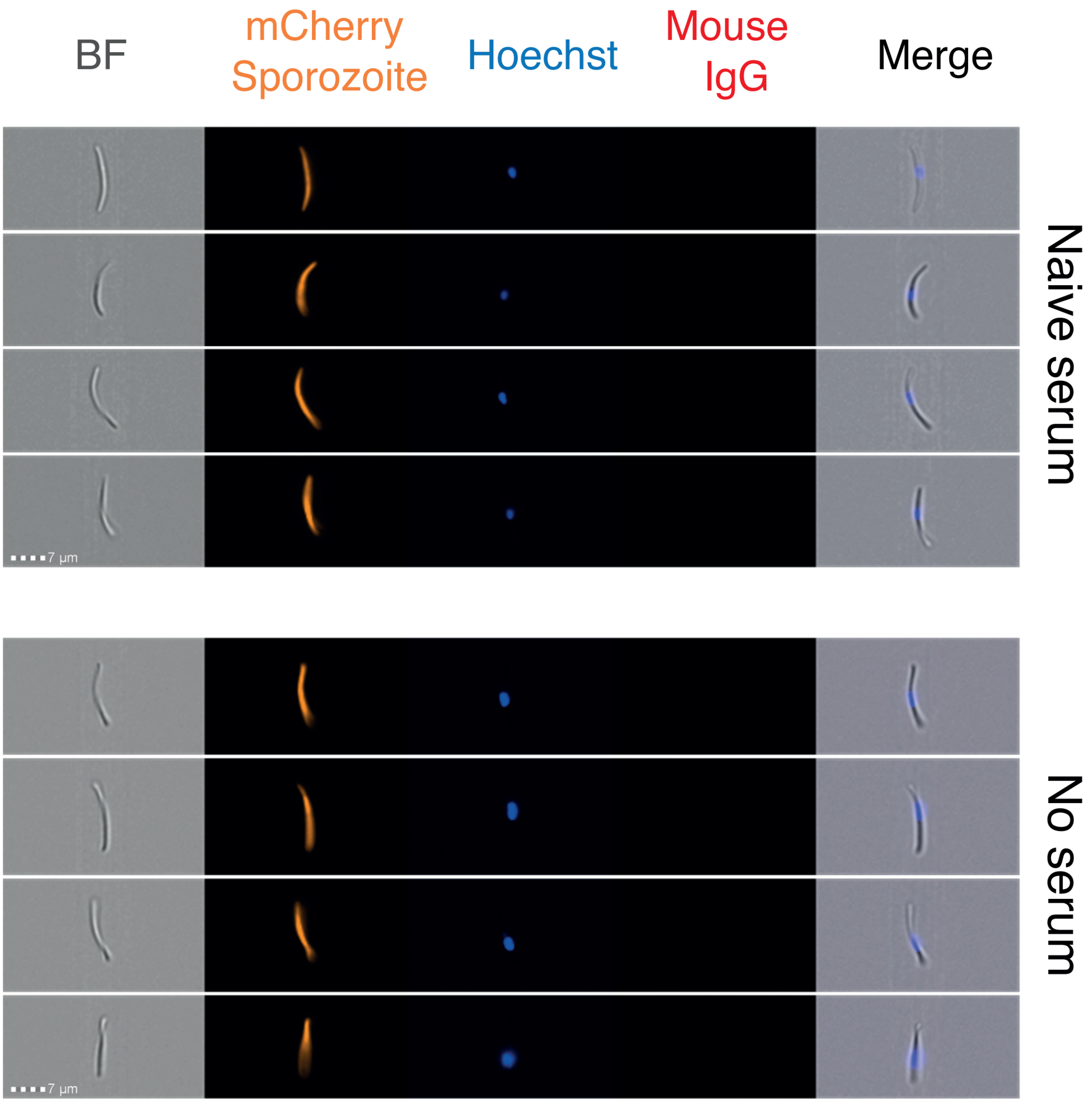

BF
mCherry Sporozoite
Hoechst
Mouse IgG
Merge
Naive serum
No serum
7 µm
7 µm

SUP FIGURE 5

A

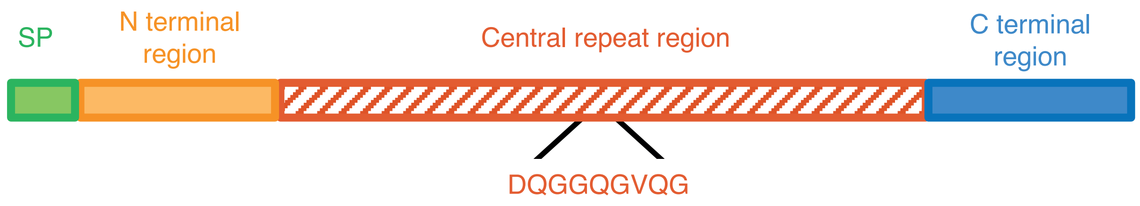

B

Predicted peptide sequence for PCHAS_0404100

MKKCTILVVASLLLVDSLLPGYGQNKNVQAQRNLNEVCYNKETDNKLYQMLNSENEKLSNRNIVNRVLADR
PDDPLDRPGDKPEDKPEDKPGDKPEDKPGDKPGDKPGDKPGDKPGDKPEDKPGDKPGDKPEDKPGDKP
GDKPGDKPGDKPADKPADKPADKPADKPADKPADKPDENKDAKKEPQHKLKQPGGQGGQGVQGDQGG
QGVQGDQGGQGVQGDQGGQGVQGDQGGQGVQGDQGGQGVQGDQGGQGVQGDQGGQGVQGDQG
GQGVQGDQGGQGGQGGQGAQGAQGGQGGQGVQGDQGGQGGQGGQGGQGGQGGQGGQGDQGAQ
GADGNRAPVVPAAPGGGGGNNNNQGGQGNQPGRGGNNNNQGGGNNNNQGGQGNQPGRGGNNNNQ
GGGNNNNQGGGNNNQGGGNNNNQGPGNQPGRGGNNNNQGGGNNNQGGGNNNNNNEEAVMPTAEHI
KEFINKIKTELTDEWSQCSVTCGTGIRIRKRAGVNKQPENYSLEDFETEVCKMDKCSSIFNIVSNSLGLVILLV
LVFFN

C

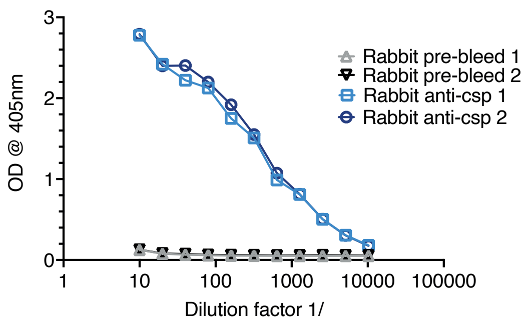

D

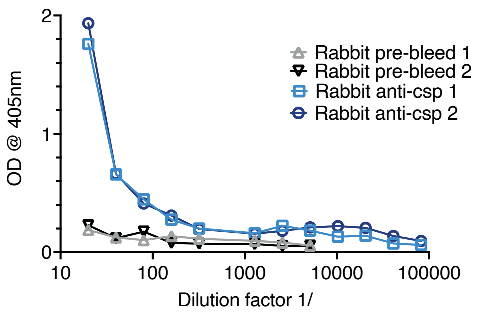

E

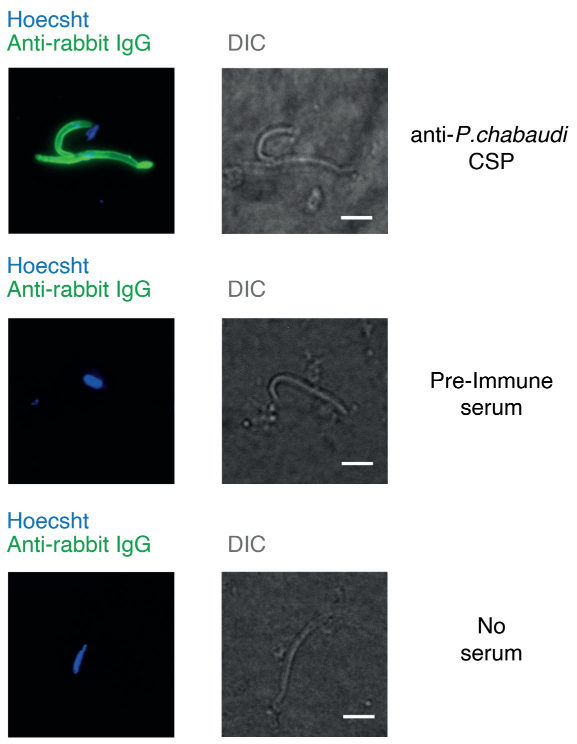

SUP FIGURE 6

IgM

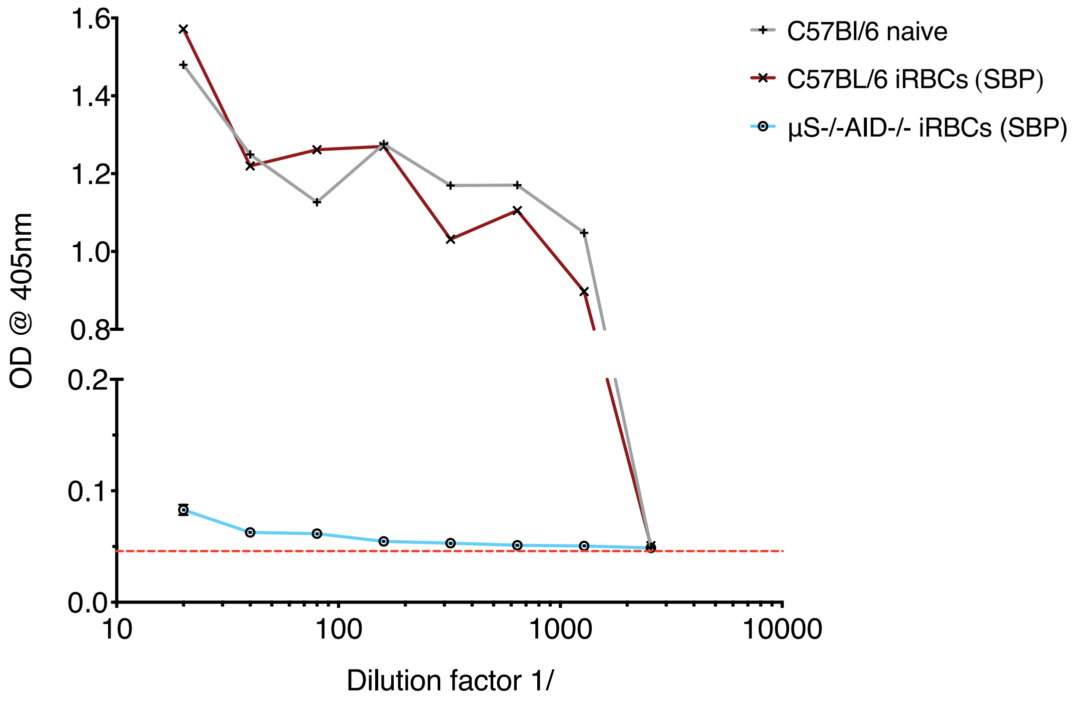

IgG

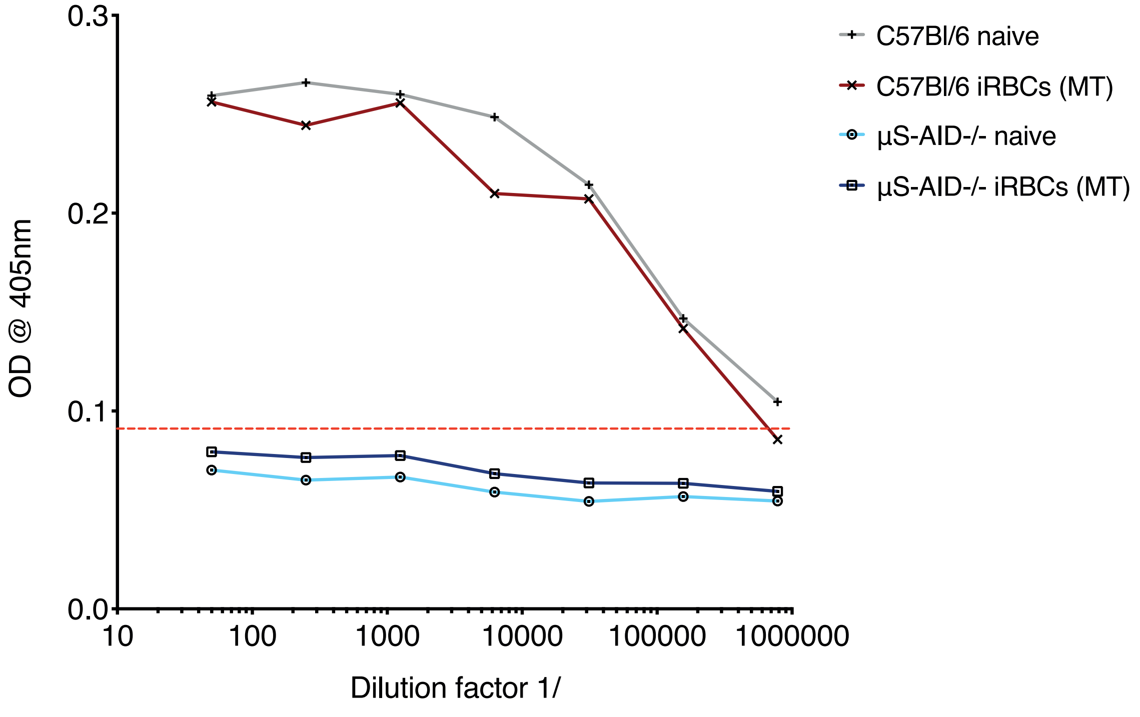

SUP FIGURE 7

A SBP infection

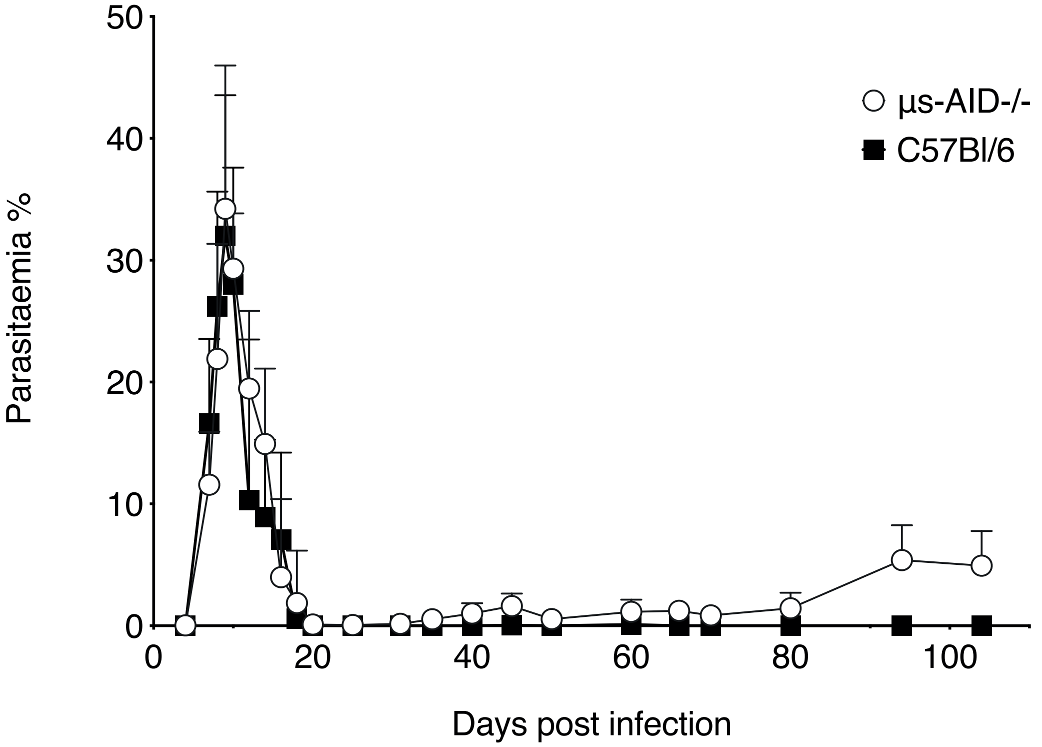

B

RMT infection

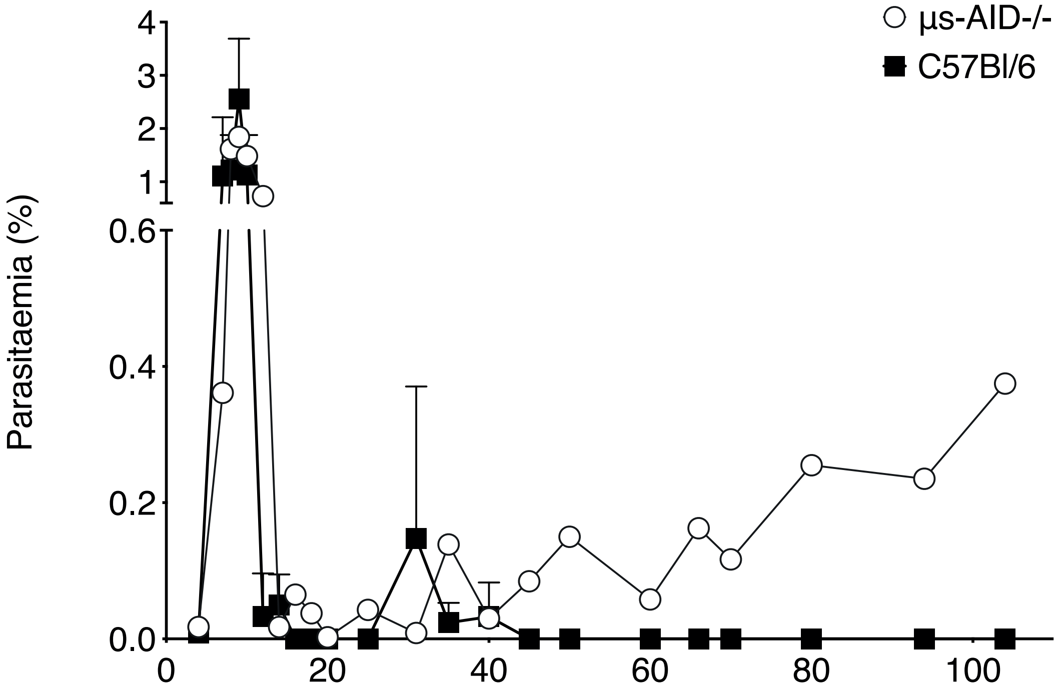

Supplement: Multimedia component 1 [file mmc1.pdf]
